# High External Quantum Efficiency Light-Emitting Diodes Enabled by Advanced Heterostructures of Type-II Nanoplatelets

**DOI:** 10.1021/acsnano.3c00046

**Published:** 2023-03-13

**Authors:** Emek G. Durmusoglu, Sujuan Hu, Pedro Ludwig Hernandez-Martinez, Merve Izmir, Farzan Shabani, Min Guo, Huayu Gao, Furkan Isik, Savas Delikanli, Vijay Kumar Sharma, Baiquan Liu, Hilmi Volkan Demir

**Affiliations:** ∥ LUMINOUS! Centre of Excellence for Semiconductor Lighting and Displays, The Photonics Institute, School of Electrical and Electronic Engineering, School of Physical and Mathematical Sciences, School of Materials Science and Engineering, Nanyang Technological University, Singapore 639798; ‡ State Key Laboratory of Optoelectronic Materials and Technologies, School of Electronics and Information Technology, Sun Yat-sen University, Guangzhou 510275, China; § Department of Electrical and Electronics Engineering, Department of Physics, UNAM—Institute of Materials Science and Nanotechnology and National Nanotechnology Research Center, Bilkent University, Ankara 06800, Turkey

**Keywords:** Type-II nanoplatelets, colloidal quantum wells, advanced heterostructures, light-emitting diodes, external quantum efficiency

## Abstract

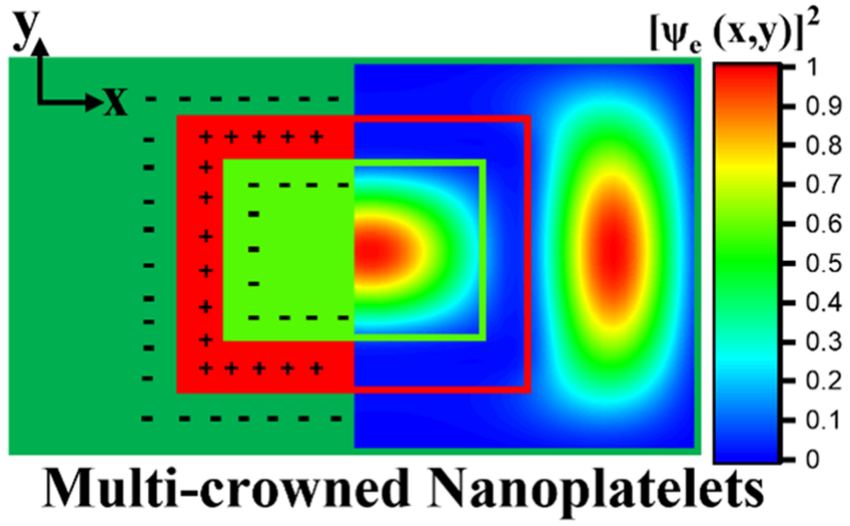

Colloidal quantum wells (CQWs), also known as nanoplatelets (NPLs), are exciting material systems for numerous photonic applications, including lasers and light-emitting diodes (LEDs). Although many successful type-I NPL-LEDs with high device performance have been demonstrated, type-II NPLs are not fully exploited for LED applications, even with alloyed type-II NPLs with enhanced optical properties. Here, we present the development of CdSe/CdTe/CdSe core/crown/crown (multi-crowned) type-II NPLs and systematic investigation of their optical properties, including their comparison with the traditional core/crown counterparts. Unlike traditional type-II NPLs such as CdSe/CdTe, CdTe/CdSe, and CdSe/CdSe_*x*_Te_1–*x*_ core/crown heterostructures, here the proposed advanced heterostructure reaps the benefits of having two type-II transition channels, resulting in a high quantum yield (QY) of 83% and a long fluorescence lifetime of 73.3 ns. These type-II transitions were confirmed experimentally by optical measurements and theoretically using electron and hole wave function modeling. Computational study shows that the multi-crowned NPLs provide a better-distributed hole wave function along the CdTe crown, while the electron wave function is delocalized in the CdSe core and CdSe crown layers. As a proof-of-concept demonstration, NPL-LEDs based on these multi-crowned NPLs were designed and fabricated with a record high external quantum efficiency (EQE) of 7.83% among type-II NPL-LEDs. These findings are expected to induce advanced designs of NPL heterostructures to reach a fascinating level of performance, especially in LEDs and lasers.

## Introduction

Semiconductor colloidal nanoplatelets (NPLs) have attracted great attention over the past decade as a promising family of optoelectronic colloidal nanocrystals. Thanks to the anisotropic shape and tight quantum confinement along the vertical direction, NPLs possess many interesting thickness-dependent optical characteristics,^1−3^ such as narrow photoluminescence (PL),^4,5^ high quantum yield (QY),^6^ giant oscillator strength,^7,8^ giant modal gain coefficient,^9−11^ and large absorption cross-section.^11,12^ Notably, unlike colloidal quantum dots, which are limited by only core@shell heterostructures, NPLs take advantage of their anisotropic 2D shape.^13−15^ With such an anisotropy, coupled with selective synthetic growth techniques, one can design a rich set of heterostructures, including but not limited to lateral extension (core/crown) or shell coating (core/shell).^1,15^

In recent years, several groups developed a variety of heterostructures to enhance the optical properties of NPLs, including improved spectral coverage,^16−18^ high QY,^6,19^ high gain,^11,20,21^ and large absorption cross-sections.^22,23^ In addition, to further enhance the optical properties, the electron and hole wave function distribution can be further manipulated by changing the material composition and geometry of these heterostructures and utilizing various band alignments of type-I and type-II.^1,13^

Previously, few different type-II NPLs, including CdSe/CdTe,^23^ CdTe/CdSe,^24^ and CdSe/CdSe_1–*x*_Te_*x*_^17^ were reported. In type-II semiconductors, the electron and hole wave functions are localized in different domains of the heterostructure.^23,25,26^ Therefore, due to the indirect charge recombination pathway at the interface between CdSe and CdTe layers, type-II NPLs exhibit substantially red-shifted emissions with a significant Stokes shift and an extended PL lifetime by several folds compared to that of type-I NPLs.^25,27^ In addition, a quasi-type-II structure was used in a few studies by employing an alloyed CdSe_*x*_Te_1–*x*_ crown to obtain enhanced optical properties such as improved QY and emission tunability compared to pristine CdSe/CdTe NPLs.^18,27^

Cd-based NPL-LEDs offer excellent prospects for display and lighting technologies owing to their attractive properties inherent from their Cd-containing NPLs such as high external quantum efficiency (EQE), high color purity, and low power consumption, in addition to their solution processability potentially enabling low-cost fabrication and flexibility.^16,28−30^ In the literature, type-I NPLs have been widely investigated for LEDs applications. In contrast, due to their reported low EQE, little attention has been paid to type-II NPL-LEDs. Lui et al. reported type-II NPL-LEDs five years ago, with an EQE of 3.57%, as the highest reported level for type-II NPLs to date.^29^ In several studies, it has been reported that large Stokes shifts in type-II NPLs favorably allow for suppressed energy transfer among their neighboring NPLs when close-packed into films and reduced reabsorption across these films, as typically prepared to be used as an electroluminescent layer in an LED.^17,29,31^

Recently, advanced heterostructures such as core/crown/crown, core@gradient-shell, core@shell@shell, and platelet-in-box have been reported to enhance the optical properties.^6,11,19,21,32,33^ For example, CdSe/CdS/CdTe core/crown/crown NPLs enable dual emission by employing a CdS barrier layer between the core CdSe and outer crown CdTe.^32^ CdSe/CdSe_1–*x*_Te_*x*_/CdS core/crown/crown heterostructures of type-II electronic band alignment are capable of exhibiting increased absorption cross-section, large gain coefficients, and reduced stacking formation.^6^ Such type-II engineered architectures of multi-crowned heterostructures hold a great potential to overcome the low device performance of conventional Cd-containing type-II NPL-LEDs, and similarly, building heterostructures in such advanced architectures of different types could potentially offer a viable means to enhance optical properties of Cd-free NPLs (made of, for example, PbS, ZnSe, and ZnTe). However, neither the advanced heterostructures of type-II NPLs nor their use in LEDs has yet been studied. Furthermore, high-performance type-II NPL-LEDs have not been reported to date.

In this work, we demonstrate the synthesis of the advanced heterostructured architecture of CdSe/CdTe/CdSe core/crown/crown NPLs with type-II band alignment. The multilayered crown synthesis was carried out using a one-pot reaction by sequential crown growth processes of CdTe and CdSe crown layers. We performed detailed optical and structural characterizations of these synthesized CdSe core, CdSe/CdTe core/crown, and CdSe/CdTe/CdSe core/crown/crown NPLs. We also investigated the type-II free carrier distribution using numerical modeling to study the electron and hole wave functions of these core/crown and multi-crown NPLs and understand the improved QY and longer lifetime of multi-crown NPLs compared to core/crown NPLs. Finally, these highly efficient multi-crowned NPLs were employed in LEDs as active material. The obtained LED emits at ∼647 nm with a high external quantum efficiency of 7.83%, a record value for NPL-LEDs based on type-II NPLs.

## Results and Discussion

In this study, we developed CdSe/CdTe/CdSe type-II core/crown/crown NPLs, investigated their optical properties, and studied the free carrier recombination mechanism in these NPLs. To synthesize these NPLs, we started with 4 monolayers (ML) thick CdSe core NPLs. Here, NPLs are defined by the number (*X*) of monolayers (ML), in which *X* represents the number of Se crystalline planes attached to the *X* + 1 planes of Cd. Thus, the NPLs possess atomically precise thickness, terminated with Cd layers at the bottom and top of the NPLs.^2^ Therefore, 4 ML CdSe NPLs consist of 4 layers of Se and 5 layers of Cd, corresponding to roughly 1.4 nm thickness (Figure S1). We used 4 ML core CdSe NPLs, as a seed to design a one-pot reaction of two-step growth of the inner CdTe crown and outer CdSe crown layers (see the Methods for details).

**Figure 1:**
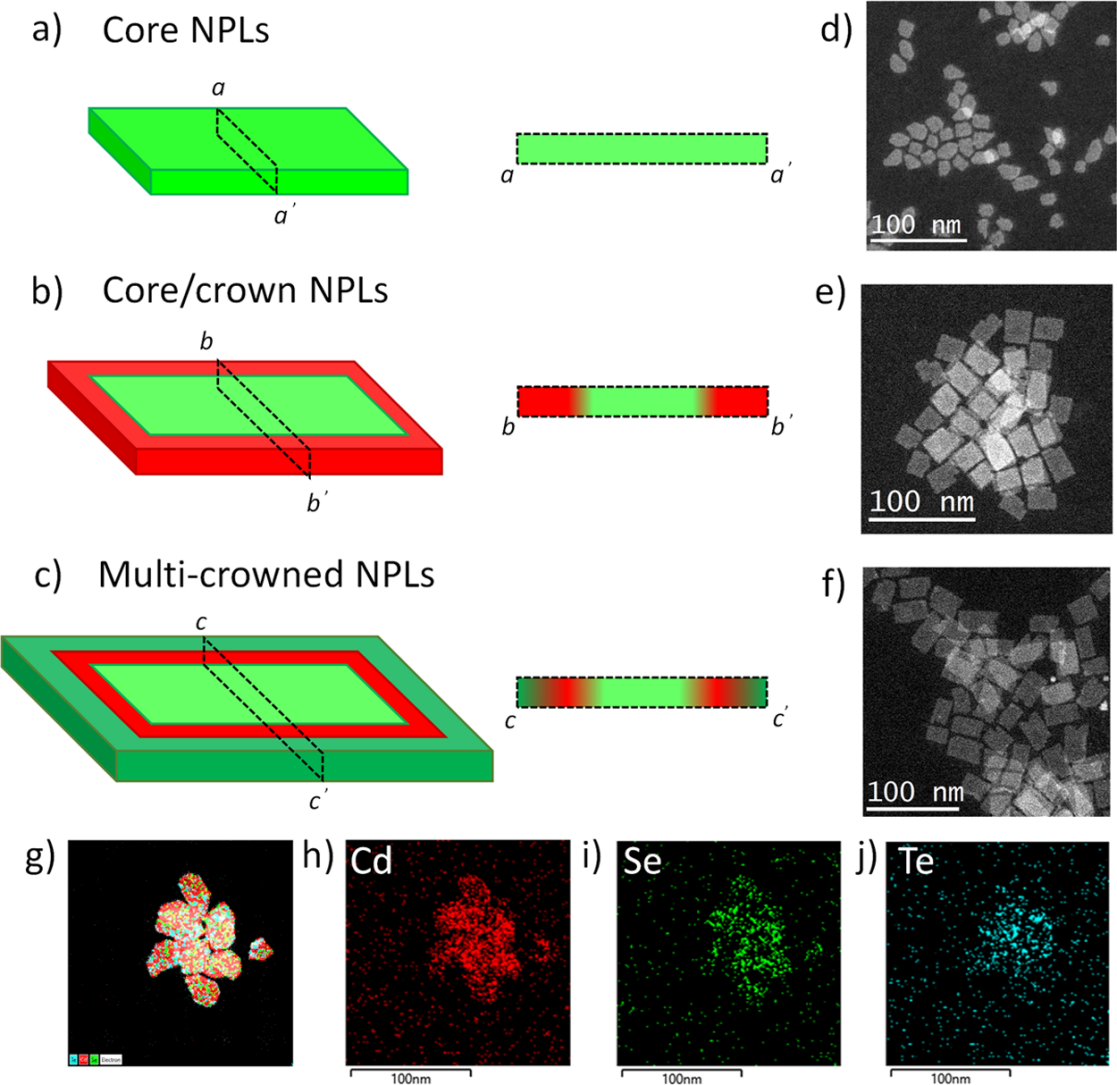
(a–c) Schematic sketch and lateral cross-sectional of views of (a) CdSe core, (b) CdSe/CdTe core/crown, and (c) CdSe/CdTe/CdSe multi-crowned NPLs. (d–f) STEM images of (d) CdSe core, (e) CdSe/CdTe core/crown, and (f) CdSe/CdTe/CdSe multi-crowned NPLs. (g–j) EDX elemental mapping spectra for all elements (g) combined and individual (h) Cd (red), (i) Se (green), and (j) Te (blue) elements of CdSe/CdTe/CdSe multi-crowned NPLs.

Figures 1a–c schematically present illustrations and cross-sectional views of CdSe (core), CdSe/CdTe (core/crown), and CdSe/CdTe/CdSe (multi-crowned) NPLs, respectively. Cross-sectional images display the interfaces between the various layers of these NPLs. These interfaces are free carrier recombination interfaces of electrons and holes localized in spatially separated layers throughout the type-II electronic alignments. As seen in the schematics, for core/crown NPLs, only a single type-II transition is possible between the CdSe core and the CdTe crown. We use this heterostructure to enable two transitions between the CdSe core–CdTe crown layers and the CdTe crown–CdSe outer crown layers (Figures 1a–c). Figures 1d–f show STEM images of the core, core/crown, and multi-crowned NPLs. As shown in these images and the size distribution histograms of length and width dimensions (Figures S2–S4), NPLs grow in lateral size after consecutive crown growth. In addition, the STEM images reveal that the core NPLs have more irregular edges, while the core/crown and multi-crowned NPLs appear with well-defined sharp edges of rectangular shape. Previously, it was reported by Abécassis et al. that NPLs with well-defined edges have a higher tendency to form superlattices.^34,35^ The TEM images show the same phenomenon of the core/crown NPLs possessing a higher tendency to form face-to-face stacks than the multi-crowned NPLs (Figures S5a–b). As previously reported, reduced stacking is essential to preventing nonradiative energy transfer along the NPL chains in films, known as FRET (Förster resonance energy transfer), to fabricate high-performance optoelectronic devices.^6,36^ Furthermore, as previously reported, we did not observe any precipitation in the nonpolar solvents for the multi-crown NPLs after long-term storage.^6,35^

Figures 1h–j show EDX mapping for Cd, Se, and Te elements for the multi-crowned NPLs. As our NPLs are very thin (∼1.4 nm) and covered by a layer of organic ligands, we could not collect a decent signal for quantitative analysis and differentiate CdTe and CdSe with the EDX method. Yet, this study confirms higher contents for Cd and Se elements than Te elements, as expected, and the elemental mapping overlaps nicely with the STEM image of NPLs. In addition to the EDX measurements, we confirmed Cd, Se, and Te elements in both core/crown and multi-crowned NPLs with the XPS analyses (Figures S6–S7).

**Figure 2:**
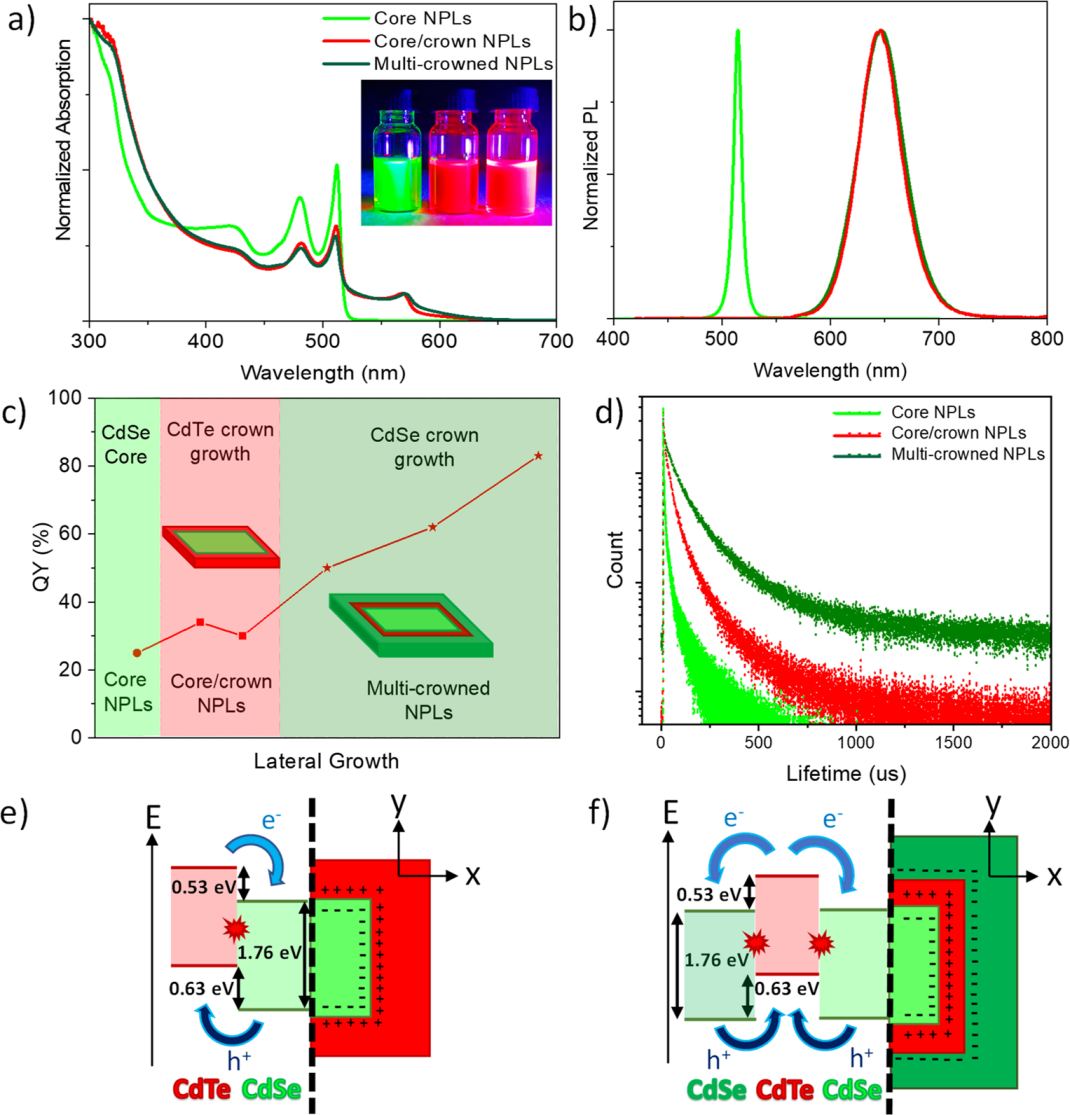
(a) Normalized absorption and (b) PL spectra of the core, core/crown, and multi-crowned NPLs. The inset picture in part a shows the emission from core, core/crown, and multi-crowned NPLs under UV lamp excitation (from left to right). (c) QY evolution of the core, core/crown, and multi-crowned NPLs throughout the crown growth processes. (d) PL decay curves of the core, core/crown, and multi-crowned NPLs at the emission peak wavelength. (e and f) Schematics of the band offsets between CdSe and CdTe layers and their respective charge transition interfaces for the (e) core/crown and (f) multi-crowned NPLs.

We performed optical characterizations of the NPLs to investigate the exciton recombination mechanism. Figures 2a–b show the absorption and emission spectra for the core, the core/crown, and the multi-crowned NPLs. Four ML thick CdSe core NPLs exhibit intrinsic well-defined optical features with two narrow absorbance peaks belonging to light hole (lh) at 480 nm and heavy hole (hh) at 512 nm and a narrow emission feature at 515 nm with a full-width at half maximum (fwhm) of 9 nm (bright green spectra in Figures 2a–b).

As the crown growth process takes place only in the lateral dimensions (length and width), this process weakly influences the quantum confinement of NPLs, as their lateral size is already larger than the 3.5–4.0 nm exciton Bohr radius of CdSe NPLs.^37,38^ Therefore, after CdTe crown growth, for CdSe/CdTe NPLs (red spectra in Figures 2a–b), the absorption features of the core NPLs do not shift, unlike the case of core/shell heterostructures with vertical growth. In addition to the lh and hh peaks of core NPLs, an additional feature arises as the CdTe layer grows, which belongs to the hh of CdTe at 570 nm.^32^ The core/crown type-II NPLs exhibit a Stokes-shifted broad emission at 645 nm with an fwhm of 47 nm. The emission mechanism completely differs for the core and core/crown heterostructures because of the conduction band (CB) and valence band (VB) levels of CdSe and CdTe. While exciton recombination occurs between the excited electrons at the CB and the holes at the VB of CdSe for the core NPLs, in the case of the type-II core/crown heterostructure the exciton recombination occurs between the excited electrons at the CdSe CB and the holes at the CdTe VB.^23,24^ This kind of indirect exciton recombination, known as charge transfer (CT), results in a large Stokes-shifted emission with a broad fwhm and a longer lifetime. Owing to this Stokes-shifted emission, type-II NPLs exhibit reduced reabsorption among NPLs. This is essential to preventing the QY loss and the nonradiative losses through FRET observed for NPL films.^36,39^ Furthermore, we investigated the evolution of the CdTe crown growth during the synthesis at different stages, which was controlled by the injected amount of the crown growth precursor (Figures S8a–b). The absorption spectrum shows that the CdTe peak does not shift, but its intensity increases with the CdTe crown’s growing size.

Similarly, there is a minor change (∼1 nm) in the emission peak wavelength, resulting from the electron wave function relaxation due to NPLs’ lateral expansion throughout the CdTe crown growth. STEM images also confirm the CdTe crown expansion (Figures S9a–c). Moreover, STEM images reveal that the edges of NPLs become sharper, and their shapes become uniform throughout the CdTe crown expansion. For the multi-crowned NPLs, the CdTe crown growth was followed by the CdSe crown. The slight change in the position of both absorption and PL features suggests that the CdSe crown growth does not alter the confinement in the heterostructure (dark green spectra in Figures 2a–b).

To investigate the type-II transitions of the multi-crowned NPLs in detail, we studied the QY and PL lifetime of the core, the core/crown, and the multi-crowned NPLs (Figures 2c–d). Figure 2c depicts the QYs of the respective aliquots throughout the CdTe and CdSe crown growth processes. As seen from the spectra, although the exciton recombination route (type-II transition) changed by CdTe crown growth, the ratio between radiative and nonradiative channels remains unchanged at around ∼30% QY (the red region in Figure 2c). On the other hand, the growth of the CdSe crown increases QY up to 83%. We intentionally choose a smaller size for the multi-crowned NPLs because large-sized NPLs tend to twist and roll to reduce their surface energy, as previously reported in the literature.^33−35^ Rolled or twisted NPLs can significantly hinder obtaining homogeneous NPL film formation, an essential requirement for fabricating high-performance optical devices.^6^

The amplitude-averaged PL lifetimes of the core, the core/crown, and the multi-crowned NPLs (Figure 2d) show that the change in the exciton recombination route (type-II indirect transition) dramatically increases the average PL lifetime of the core/crown NPLs (46.2 ns) compared to the core NPLs (8.0 ns). The PL lifetimes of the core and the core/crown NPLs match the previously reported lifetimes.^6,32^ For the multi-crowned NPLs, the PL lifetime elongated to 73.3 ns, which is consistent with the QY increase compared to the core/crown NPLs. Such an increase in the QY, accompanied by the increase in the PL lifetime on the multi-crowned NPLs, can be attributed to the suppression of nonradiative recombination channels by adding a CdSe crown. The PL lifetime comparison supports our observations with absorption, PL, and QY characterizations (Figure 2d, Table S1).

The optical investigations suggest that the core/crown and multi-crowned NPLs possess different exciton recombination mechanisms than the core NPLs originating from the band alignment of CdSe and CdTe. Figures 2e–f illustrate the band offsets between CdSe and CdTe layers and their respective charge transition interfaces for the core/crown and the multi-crowned NPLs (see the theoretical section in the Supporting Information for details). As a typical type-II alignment, in the core/crown NPLs, the conduction band offset of 0.53 eV between CdTe and CdSe allows only photogenerated electrons to move to the CB of the CdSe core. On the other hand, the valence band offset of 0.63 eV causes the hole carriers to migrate to the VB of the CdTe crown.^32^ The Stokes shift broadened emission results from an indirect transition between the CB of CdSe and the VB of CdTe, which happens at the interface of the CdSe and CdTe layers (Figure 2e). As band offsets are an intrinsic property of the material combinations, for these multi-crowned NPLs, we expect that the band offsets between the CdSe crown and the CdTe crown are identical to those between the CdTe crown and the CdSe core (Figure 2f). As there are two interfaces in the multi-crowned NPLs between the CdSe and CdTe layers, we can presume the possibility of an additional type-II transition to occur between the CdTe crown and the CdSe crown.

To develop additional insights on the type-II transition, we studied the ultrafast charge dynamics of our heterostructures with transient absorption by exciting them with a 400 nm fs-laser (Figures S10–S11). For both the core/crown and the multi-crowned NPLs, we observed bleaches (ΔA), indicating state-filling of CB levels at 480, 512, 565, and 630 nm related with CdSe-lh, CdSe-hh, CdTe, and CT, respectively.^25^ As we observed CT bleach, TA confirms type-II band alignment for both heterostructures. Furthermore, the TA study of the core/crown and the multi-crowned NPLs suggests that charge recombination is more effective for the multi-crowned compared to the core/crown NPLs, as the decay is faster for the CdSe-hh and CdTe features for the multi-crowned (Figure S11). Therefore, it is logical to presume that by enabling two type-II transitions, our advanced multi-crowned design favors more efficient charge recombination.

**Figure 3:**
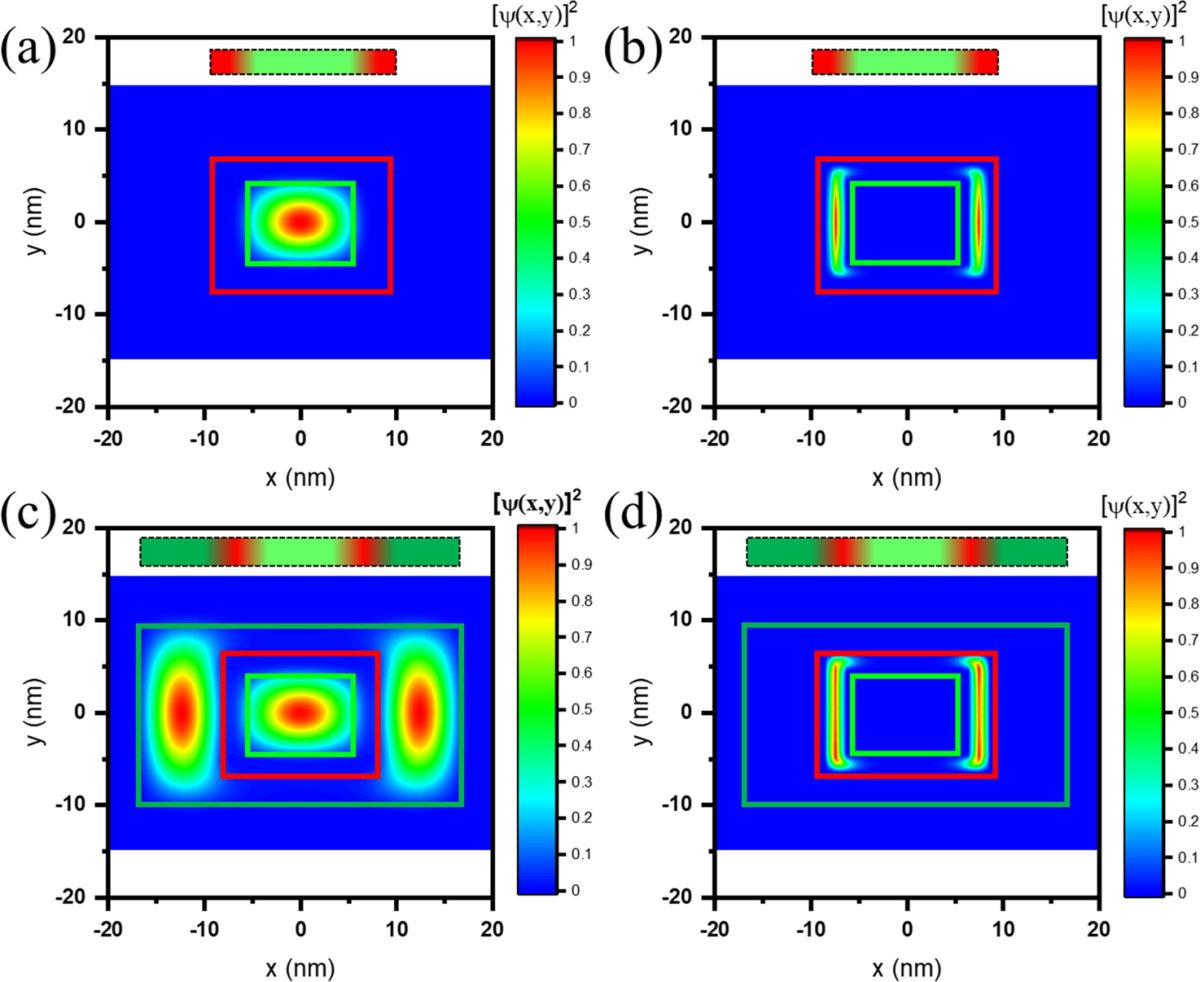
(a and c) Electron wave function for (a) the core/crown and (c) multi-crowned NPLs. (b and d) Hole wave function for (b) the core/crown and (d) multi-crowned NPLs. The core, core/crown, and multi-crown layers are indicated as bright green, red, and dark green rectangles, respectively, to guide the eye and for illustration purposes.

We numerically simulated the carrier wave function distributions in our NPL heterostructures to confirm our hypothesis of the exciton recombination mechanism in the multi-crowned NPLs, the results of which are shown in Figures 3a–d (see the Theoretical Section in the Supporting Information for details). The calculations show that, in the case of the core/crown NPLs, the electron wave function remains confined at the CdSe core (bright green rectangle, Figure 3a).^31,40^ In the case of multi-crowned NPLs, the electron is delocalized in both the CdSe core and the CdSe crown layers (dark green rectangle, Figure 3c). In the case of the hole wave function, it remains confined at the CdTe crown for both the core/crown and the multi-crowned NPLs (red rectangle, Figures 3b–d). However, it is worth noting that the hole wave function of the multi-crowned NPLs is distributed uniformly among the CdTe crown (Figure 3d), which expands to its corners and edges compared to the core/crown NPLs case (Figure 3b). According to the insights obtained from the computations, the multi-crowned NPLs have two significant advantages compared to the core/crown NPLs: (i) The more uniform distribution of the hole wave function can/may improve electron–hole recombination, and (ii) multi-crowned NPLs having an electron wave function distributed in both the CdSe core and the CdSe crown layers will favor the two type-II transitions from the CdSe core to the CdTe crown and from the CdSe crown to the CdTe crown.

**Figure 4:**
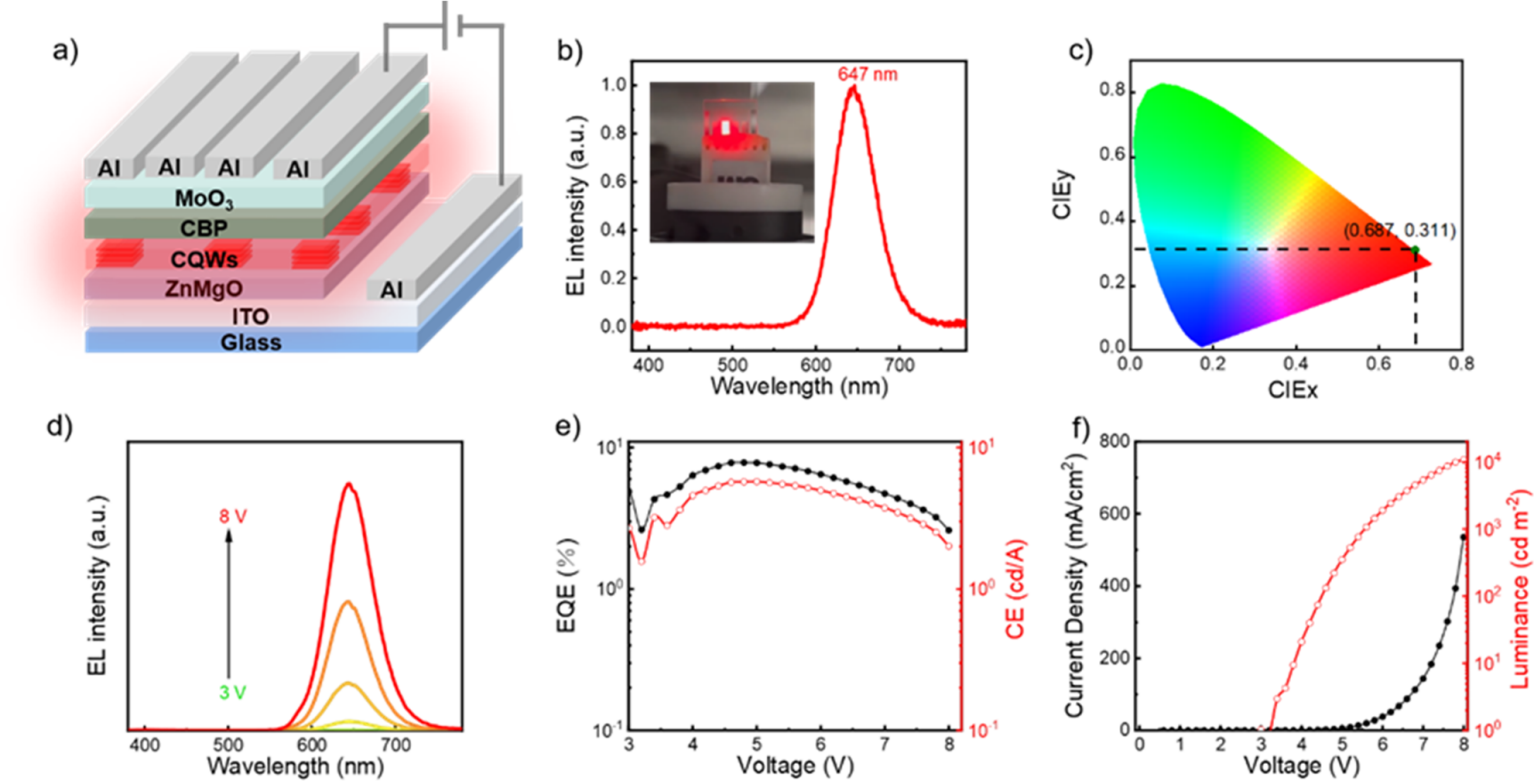
(a) Device structure of the multi-crowned NPL-LEDs. (b) EL spectrum of the multi-crowned NPL-LEDs. Inset is a photograph of NPL-LEDs under bias. (c) Position of the coordinates of (0.687, 0.311) in the CIE diagram. (d) EL spectra of the multi-crowned NPL-LEDs under different biases varied in the steps of 1 V. (e) EQE and CE of the multi-crowned NPL-LEDs. (f) Current density and luminance of the multi-crowned NPL-LEDs.

Up to here, we have investigated structural and optical characterizations of the proposed multi-crowned NPLs and presented their free carrier wave function distribution calculations. Based on their high QY, less tendency to stacking, and reduced reabsorption owing to their Stokes shifted emission, the type-II multi-crowned NPLs are very suitable materials for NPL-based LEDs. To explore the EL properties of these NPLs, we fabricated NPL-based LEDs using our multi-crowned NPLs. These NPL-LEDs are structured with an inverted hybrid organic–inorganic architecture as shown in Figure 4a: indium tin oxide (ITO, 135 nm)/Mg-doped zinc oxide (ZnMgO, 30 nm)/CdSe/CdTe/CdSe type-II core/crown/crown NPLs (30 nm)/4,4-N,N-dicarbazolebiphenyl (CBP, 45 nm)/molybdenum trioxide (MoO_3_, 6 nm)/Al (60 nm), where NPLs were used as the emitting layer (EML). NPLs were cleaned to reduce the excess ligands and then dispersed in hexane to facilitate spin-coating onto the ZnMgO electron transporting layer (ETL) without dissolution. CBP and MoO_3_ serve as the hole-transporting layer (HTL) and the hole-injecting layer (HIL), respectively. ITO and Al function as the cathode and anode contacts, respectively. The cross-sectional image of the resulting LED device is presented in Figure S12. Since CBP possesses the highest occupied molecular orbital (HOMO) of 5.9 eV and a high hole mobility of 1 × 10^–3^ cm^2^ V^–1^ s^–1^, holes can be effectively transported.^29^ Meanwhile, ZnMgO ensures the electron injection due to the matched conduction band of 3.9 eV with NPLs and suitable electron mobility.^41^ Therefore, such a device structure of NPL-LEDs can have a balanced injection of electrons and holes into the NPLs, assuring high performance.

Figures 4b–f show the properties of NPL-LEDs with the multi-crowned NPLs as EML. As shown in Figures 4b–c, the EL emission peak wavelength of NPL-LEDs is 647 nm, corresponding to the CIE coordinates of (0.687, 0.311). The EL spectrum shows a stable emission with no change in the EL peak wavelength with increasing voltage (from 3 to 8 V) (Figure 4d). To the best of our knowledge, the previously highest reported EQE for the type-II NPL-LEDs was 3.57% (Table S2).^29^ Our multi-crowned NPL-LEDs exhibited the maximum EQE of 7.83% (Figure 4e), a record for LEDs with type-II NPLs. The maximum current efficiency (CE) of the multi-crowned NPL-LEDs is 5.74 cd A^–1^ (Figure 4e). In addition, the maximum luminance of the multi-crowned NPL-LEDs is 10,765 cd m^–2^, and the turn-on voltage (the voltage at which the luminance is >1 cd m^–2^) is 3 V (Figure 4f). As a comparison, NPL-LEDs using core/crown NPLs exhibit poor device properties (e.g., the maximum EQE is 0.77%, Figure S13), similar to previous attempts.^29^ The reason for the high performance of the multi-crown NPL-LEDs is the higher QY of the advanced heterostructure, the homogeneity of the multi-crown NPL films (see atomic force microscopy (AFM) results shown in Figure S14), the favorable electron–hole migration, the good electron–hole recombination, and a proper band alignment of all device layers. In addition, this hybrid device architecture can provide sufficient electrons and holes to reach the NPL-based EML and then generate excitons for emission since ZnMgO possesses good surface morphology, suitable charge injection, and charge transfer capability, which is suitable with the hole injection of CBP.^41^ These findings indicate that the multi-crowned NPLs enable high-performance solution-processed LEDs, which may offer a great potential to empower next-generation NPLs-based display and lighting technologies.

## Conclusions

In conclusion, we have established the synthesis of an advanced heterostructure of CdSe/CdTe/CdSe multi-crowned NPLs. The designed multi-crowned NPLs possess type-II band alignment, leading to a red shift in emission with a broader fwhm than that of the core NPLs. Owing to the Stokes-shifted emission and longer PL lifetime due to indirect charge recombination, the multi-crowned NPLs exhibit reduced reabsorption. Furthermore, the CdSe crown improved surface passivation and reduced stacking among NPLs. Thus, we obtained the highest QY of 83% for pristine type-II NPLs. To support our hypothesis that the second type-II exciton recombination pathway increases the QY in the multi-crown heterostructure, we computed electron and hole wave functions for the core/crown and the multi-crowned NPLs. Numerical calculations confirmed an electron wave function distributed in both the CdSe core and the CdSe crown, along with a more uniformly distributed hole wave function at the CdTe crown for the multi-crowned NPLs, favoring better charge recombination and enabling two type-II transitions. In addition, NPL-LEDs based on the core/crown and multi-crowned NPLs were fabricated to explore their EL performances. The multi-crowned NPLs exhibit a record EQE of 7.83% and a luminance level of 10765 cd m^–2^, which are 1 order of magnitude higher than those of core/crown NPL-based LEDs. These results demonstrate the outstanding properties of such an advanced heterostructure of NPLs and their potential in NPLs-based display and lighting technologies. To further increase the performance these NPL-LEDs, it is critical to improve the morphology of the emissive NPL layer (uniformity of the NPL film thickness, controlling the orientation of NPLs, close-packing of NPLs, avoiding pinholes in the NPL film, etc.) and to engineer the interfaces of charge transport and injection layers with this NPL film for the optimization of charge injection, possibly offering great promise as a high-performance lighting and display platform.

## Methods

### Materials

Cadmium oxide (CdO) (99.9%), cadmium acetate dihydrate (Cd(OAc)_2_·2H_2_O) (>98%), myristic acid (>99%), 1-octadecene (ODE, technical-grade), tellurium (Te), selenium (Se) (99.99% all are trace metals basis), technical-grade oleic acid (OA) (90%), trioctylphoshine (TOP), hexane, and ethanol (EtOH) were purchased from Sigma-Aldrich. ZnMgO nanoparticles were purchased from Poly OptoElectronics Co. Ltd.

### Synthesis of 4 Monolayer (ML) CdSe Core NPLs

In a typical 4 ML CdSe NPL synthesis, 77 mg of CdO, 340 mg of myristic acid, and 28 mL of ODE were mixed in a 50 mL round-bottom flask, and the solution was degassed at 100 °C. Then, the solution was heated up to 285 °C under argon flow until becoming colorless and transparent and cooled down to 90 °C. In another flask, 24 mg of Se powder was dissolved in 2 mL of ODE using ultrasonication. At 90 °C, the Se-precursor solution was injected into the Cd-precursor solution, and the temperature was set to 235 °C. After the temperature reached ∼195 °C, 160 mg of Cd(OAc)_2_·2H_2_O was added swiftly. At 235 °C, the reaction was maintained for 10 min and was terminated with the addition of a 1 mL OA injection. The NPL solution was cooled down to room temperature by using a water bath, and 5 mL of hexane was injected to increase the solubility of NPLs. The obtained solution was washed with ethanol at 6,000 rpm for 5 min, and precipitates were redissolved in hexane.

### Synthesis of CdSe/CdTe Core/Crown and CdSe/CdTe/CdSe Core/Crown/Crown NPLs

In a 50 mL round-bottom flask, 1 mL of CdSe NPL solution (optical density: 1 at 350 nm for 100 μL core NPLs in 3 mL of hexane), 30 mg of Cd(OAc)_2_·2H_2_O, 0.45 μL of OA, and 4 mL of ODE were mixed with a magnetic stirrer and degassed at 100 °C. Then, under argon flow, the temperature was increased to 215 °C, and at this temperature, 0.03 M TOP-Te solution in ODE was injected at a rate of 4 mL/h by using a syringe pump. The CdTe absorption peak was tracked to monitor the growth of the CdTe crown layer via UV–vis, and after the desired CdTe crown growth was obtained, the injection was stopped. For CdSe/CdTe core/crown NPLs, following the growth of the CdTe layer, the reaction was cooled to room temperature using a water bath, and 3 mL of hexane was injected. Products were washed with EtOH addition and centrifugation at 6,000 rpm for 5 min. Precipitates were redissolved in hexane. For CdSe/CdTe/CdSe core/crown/crown NPLs, following the growth of the CdTe layer, the crown growth precursor was changed to 0.03 M TOP-ODE-Se solution under identical conditions, and injection was maintained until the desired lateral size for the outer CdSe layer was obtained. After the targeted CdSe crown growth was completed, the reaction was terminated and washed by following the same procedures as for CdSe/CdTe core/crown NPLs.

### NPL-LEDs Device Fabrication

NPL-LEDs were fabricated on patterned ITO glass substrates, and these were cleaned in an ultrasonic bath sequentially using detergent, isopropyl alcohol, and deionized water. ZnMgO layers were spin-casted from ethanol dispersions of ZnMgO nanoparticles onto cleaned ITO glass substrates at 3,000 rpm for 45 s and dried in an N_2_ atmosphere at 150 °C for 15 min. Next, the NPLs precursor solution was spin-coated on the electron transport layer (ETL) film at 2,000 rpm for 40 s. Finally, CBP, MoO_3_, and Al, in this order, were deposited on top of the NPLs film using a shadow mask by thermal evaporation. The pixel size is 8 mm^2^, which is the overlapping region between the ITO and Al electrodes.

### Optical Characterizations

UV–Vis absorption and PL spectra of NPLs were obtained using a Shimadzu UV-1800 spectrophotometer and a Shimadzu RF-5301 PC spectrofluorophotometer. For QY measurements, samples were excited with a 405 nm laser in an integrating sphere, and the data was collected with an Ocean Optics S4000 spectrometer. A time-correlated single-photon counting system with a time resolution down to 4 ps (PicoHarp 300) was used to deliver laser pulses at an 80 MHz repetition rate. This system consists of a picosecond pulsed laser with an output photon energy of 3.31 eV (375 nm) driven by a driver module (PDL-800 series) and a fast photomultiplier tube (Hamamatsu H5783 series) to resolve the lifetimes on the order of a few picoseconds. Transient absorption (TA) spectroscopy was performed to study the carrier dynamics of the samples by using a Helios setup (Ultrafast Systems LLC) and in transmission mode with chirp correction. The white light continuum probe beam (400–800 nm) was generated from a 3 mm sapphire crystal using an 800 nm pulse from the regenerative amplifier. The pump beam spot size was ∼50 μm. The probe beam passing through the sample was collected using a detector for the ultraviolet–visible region (CMOS sensor). All measurements were performed at room temperature in solution.

### Structural and Elemental Characterizations

To characterize the dimensions of the multi-crowned NPLs, we used a JEOL TEM 2100F transmission electron microscope operated at 200 kV in the high-angle annular dark-field scanning transmission electron microscopy (STEM) configuration embedded with an energy-dispersive X-ray spectroscopy (EDX) detector. X-ray photoelectron spectroscopy (XPS) measurements were performed using a Shimadzu Kratos AXIS Nova XPS instrument to analyze the elemental compositions of NPLs. The samples were spin-coated on the silicon substrates. The XPS spectra were analyzed by using the Kratos software. All peaks in the XPS spectra are corrected to the C 1s peak (285 eV).

### Device Characterization

The current density–voltage–luminance curves, electroluminescence (EL) spectra, and current efficiency (CE) and EQE measurements of the NPL-LEDs were captured using an external quantum efficiency measurement system (C9920-12, Hamamatsu Photonics Co. Ltd.).

## Supporting Information Available

Additional STEM images, size distributions, UV, PL, TRF, XPS, EL, and TA spectra, SEM and AFM images, and theoretical section for CdSe core, CdSe/CdTe crown, and CdSe/CdTe/CdSe multi-crowned NPLs. (PDF)
