# Supplementary material for: High External Quantum Efficiency Light-Emitting Diodes Enabled by Advanced Heterostructures of Type-II Nanoplatelets
Source: ACS Nano. 2023 Mar 13;17(8):7636–44. doi: 10.1021/acsnano.3c00046 (PMC10134493; doi:10.1021/acsnano.3c00046)
Supplement: Supplementary file 1 — nn3c00046_si_001.pdf [file nn3c00046_si_001.pdf]

Supplementary Information

High External Quantum Efficiency Light-Emitting Diodes Enabled by Advanced Heterostructures of Type-II Nanoplatelets

Emek G. Durmusoglu[a,‡], Sujuan Hu[b,‡], Pedro Ludwig Hernandez-Martinez[a], Merve Izmir[a], Farzan Shabani[c], Min Guo[b], Huayu Gao[b], Furkan Isik[c], Savas Delikanli[c], Vijay Kumar Sharma[a], Baiquan Liu[b], and Hilmi Volkan Demir[a,c*]*

[a] LUMINOUS! Centre of Excellence for Semiconductor Lighting and Displays, The Photonics Institute, School of Electrical and Electronic Engineering, School of Physical and Mathematical Sciences, School of Materials Science and Engineering, Nanyang Technological University, Singapore 639798

[b] State Key Laboratory of Optoelectronic Materials and Technologies, School of Electronics and Information Technology, Sun Yat-sen University, Guangzhou, 510275, China

[c] Department of Electrical and Electronics Engineering, Department of Physics, UNAM − Institute of Materials Science and Nanotechnology and National Nanotechnology Research Center, Bilkent University, Ankara 06800, Turkey

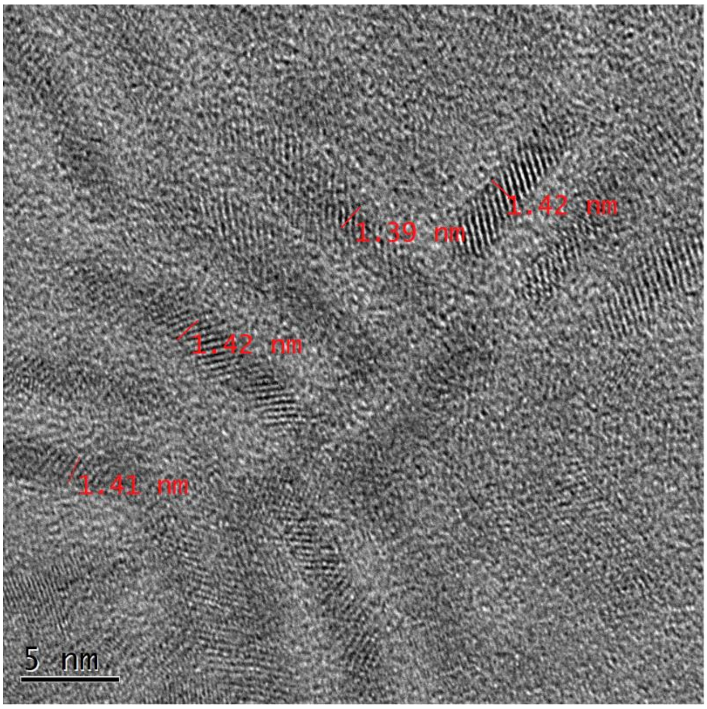

Figure S1. Transmission electron microscopy image of vertically aligned 4 ML core CdSe NPLs with their thickness labels.

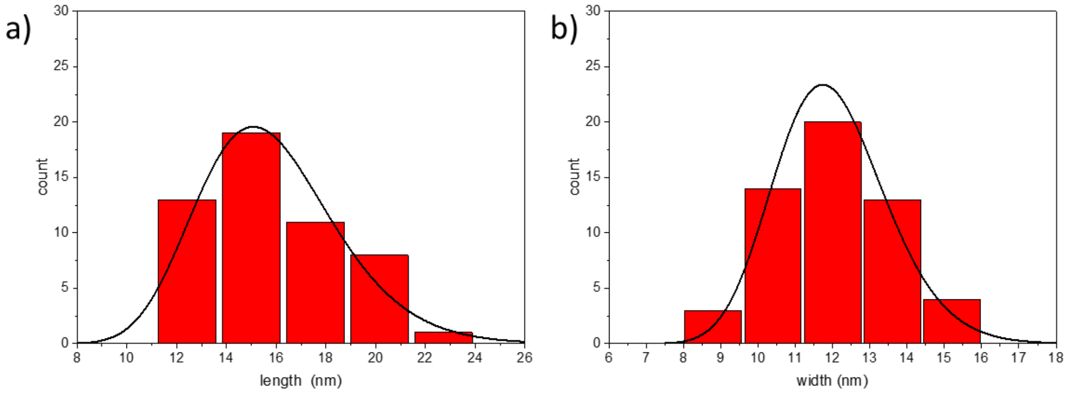

Figure S2. Size distributions of a) length and b) width dimensions of the CdSe core NPLs.

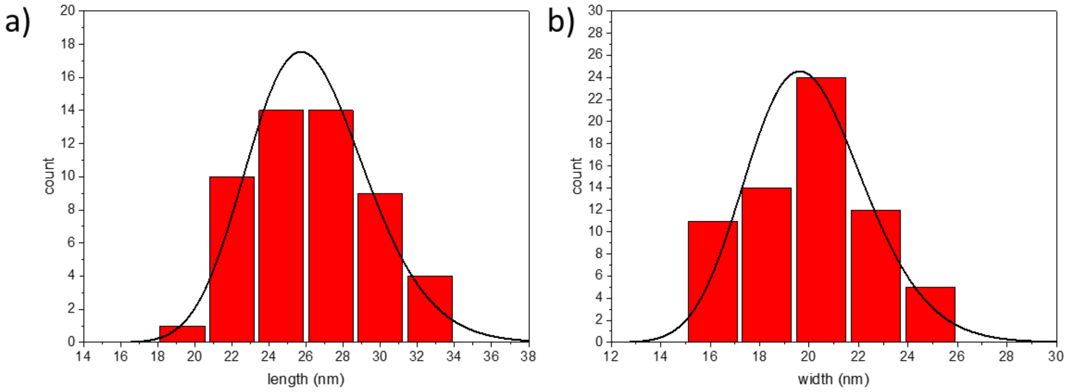

Figure S3. Size distributions of a) length and b) width dimensions of the CdSe/CdTe core/crown NPLs.

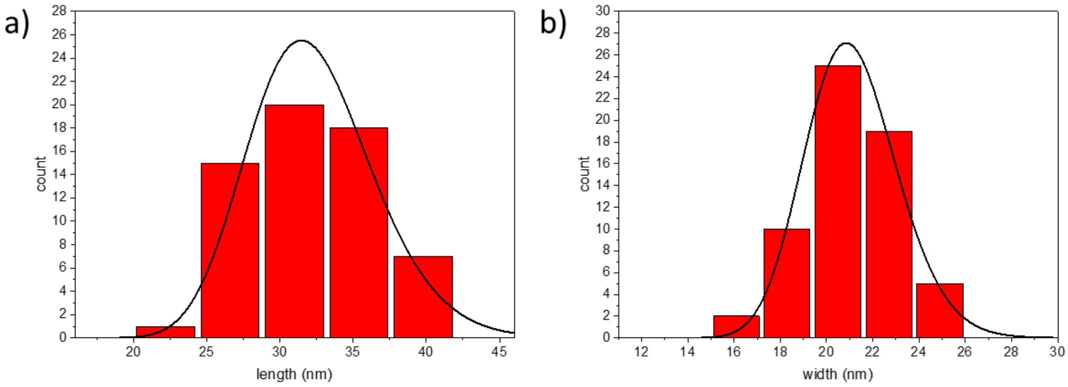

Figure S4. Size distributions of a) length and b) width dimensions of the CdSe/CdTe/CdSe core/crown/crown NPLs.

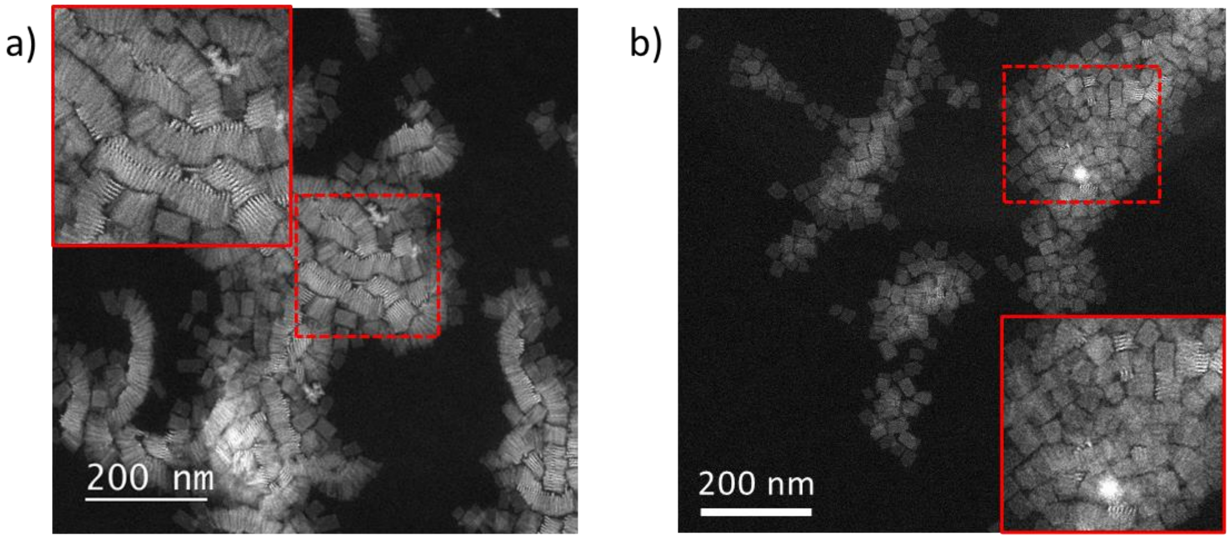

Figure S5. Large-area STEM images for a) the core/crown and b) multi-crowned NPLs. STEM images reveal that core/crown NPLs have a higher tendency to form long face-to-face NPL chains. The inset image is a magnified image of the indicated region with red rectangles.

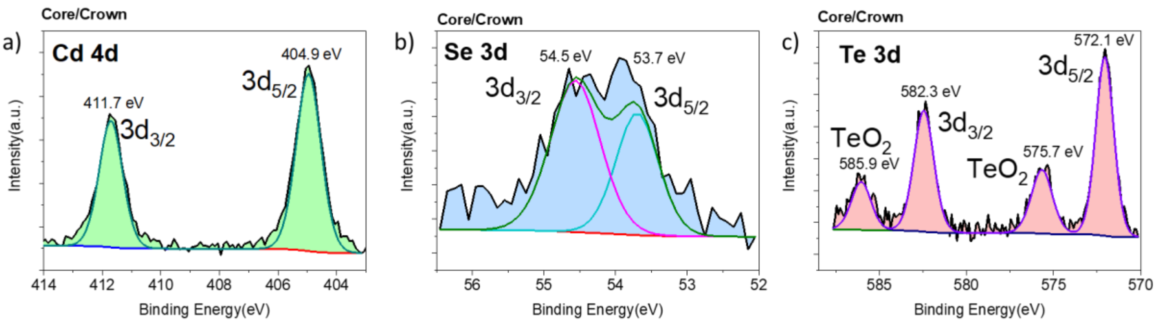

Figure S6. XPS spectra of a) Cd 3d, b) Se 3d, and c) Te 3d core levels of the core/crown NPLs.

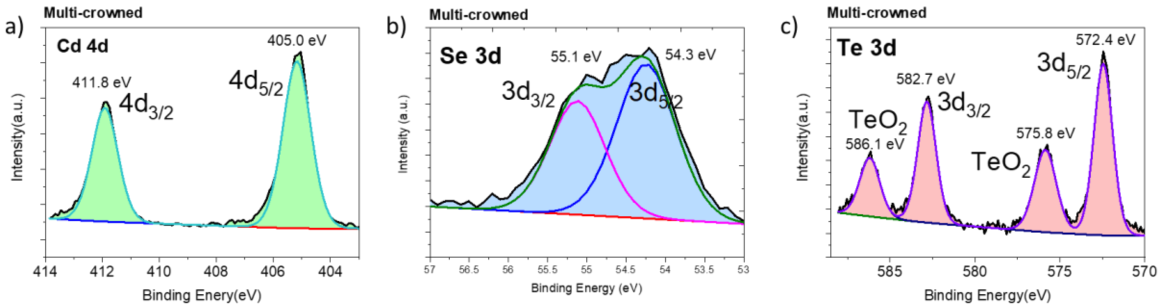

Figure S7. XPS spectra of a) Cd 3d, b) Se 3d, and c) Te 3d core levels of the multi-crowned NPLs.

X-ray photoemission spectroscopy (XPS) spectra of the core/crown and multi-crowned NPLs were investigated to determine the elemental composition of the samples. All recorded XPS spectra have been corrected to C 1s peak at 285.0 eV.

For core/crown, in Figures S6 a-c, the Cd $3d_{5/2}$ and $3d_{3/2}$ states appear at 404.9 and 411.7 eV. The Se $3d_{5/2}$ and $3d_{3/2}$ states place at 53.7 eV and 54.5 eV. Lastly, the Te $3d_{5/2}$ and $3d_{3/2}$ states present two contributions appearing at 572.1 eV and 582.3 eV.

For multi-crowned, In Figures S7 a-c, the Cd $3d_{5/2}$ and $3d_{3/2}$ states appear at 405.0 and 411.8 eV. The Se $3d_{5/2}$ and $3d_{3/2}$ states place at 50.5 eV and 51.4 eV. Lastly, the Te $3d_{5/2}$ and $3d_{3/2}$ states present two contributions at 572.4 eV and 582.7 eV.

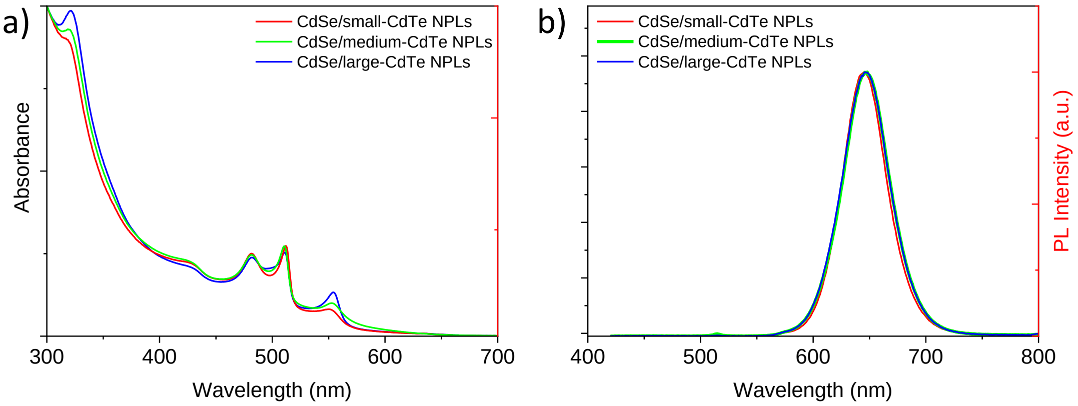

Figure S8. Evolution of a) absorption and b) photoluminescence spectra of CdSe/CdTe core/crown NPLs as CdTe crown grows.

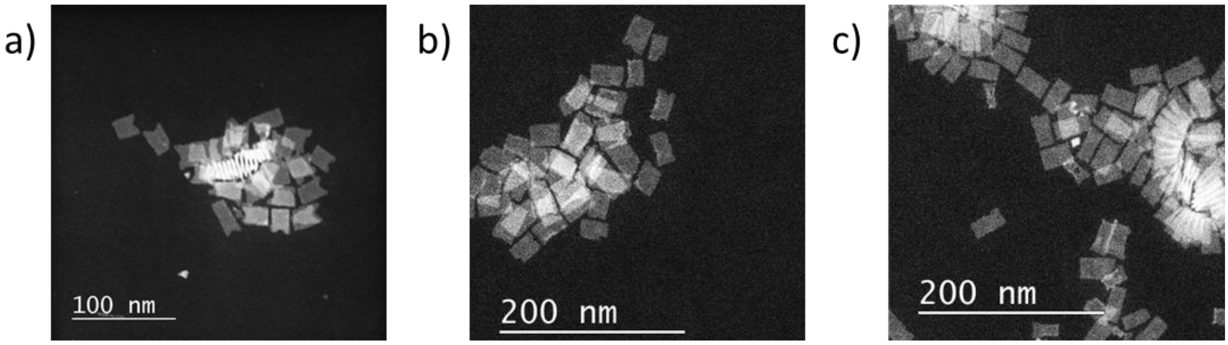

Figure S9. STEM images reveal crown growth progress among the CdSe NPLs with small-, medium-, and large crowns, respectively.

Table S1. Amplitude-averaged PL lifetime comparison of the core, core/crown, and multi-crowned NPLs at their emission peak maxima.

Sample	α_1	τ_1 (ns)	α_2	τ_2 (ns)	α_3	τ_3 (ns)	τ_{av} (ns)
**Core NPLs**	2474	2.60	566	17.05	75	118.28	8.02
**Core/Crown NPLs**	2052	15.07	1240	74.06	101	338.4	46.2
**Core/Crown/Crown NPLs**	1446	5.46	1587	83.27	339	316.2	73.3

PL decay curves were fitted with three exponentials. PL lifetimes were calculated with the following relation;

$$\langle \tau_{av} \rangle = \frac{\sum \alpha_j \tau_j}{\sum \propto_j}, \quad \text{(S1)}$$

where τ_{av} is the amplitude-averaged PL lifetime, α is the amplitude, and τ is the lifetime of a component.

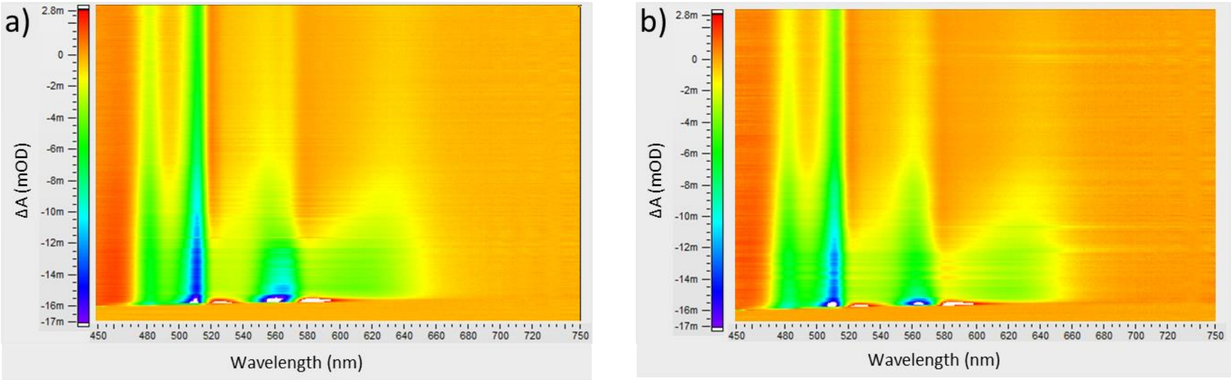

Figure S10. Transient absorption mapping of a) the core/crown NPLs and b) the multi-crowned NPLs at 50 μJ/cm^2 pump fluence.

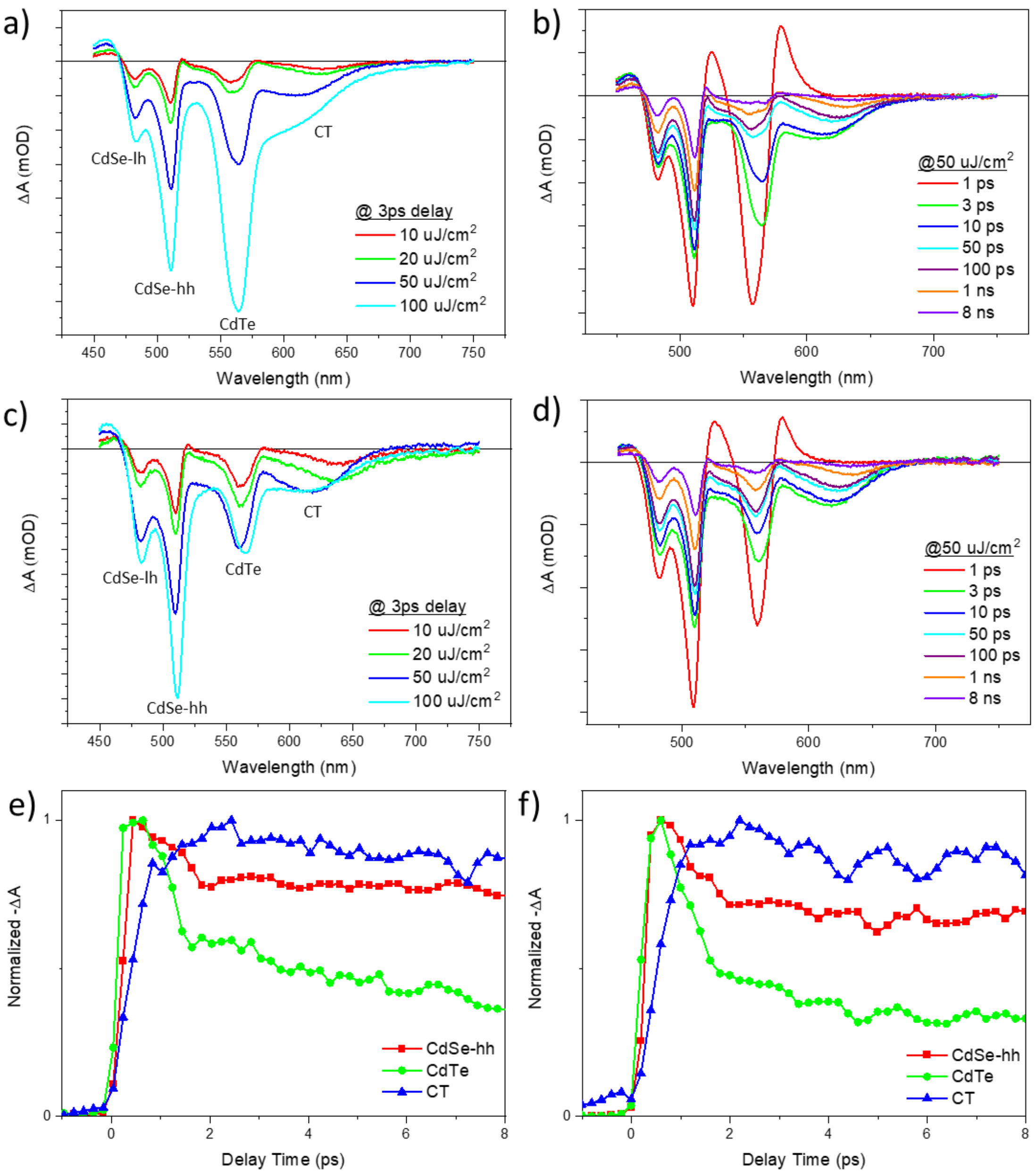

Figure S11. Transient absorption (TA) spectra of a) the core/crown and c) multi-crowned NPLs show the change in ΔA with an increasing pump fluence after a 3 ps delay. TA spectra of b) the core/crown and d) multi-crowned NPLs show the change in ΔA with an increasing delay at 50 μJ/cm^2 pump fluence. ΔA versus delay time spectra of e) core/crown and f) the multi-crowned NPLs show transient decay at CdSe-hh, CdTe, and CT states at 50 μJ/cm^2 pump fluence. The excitation wavelength is 400 nm for all of the measurements.

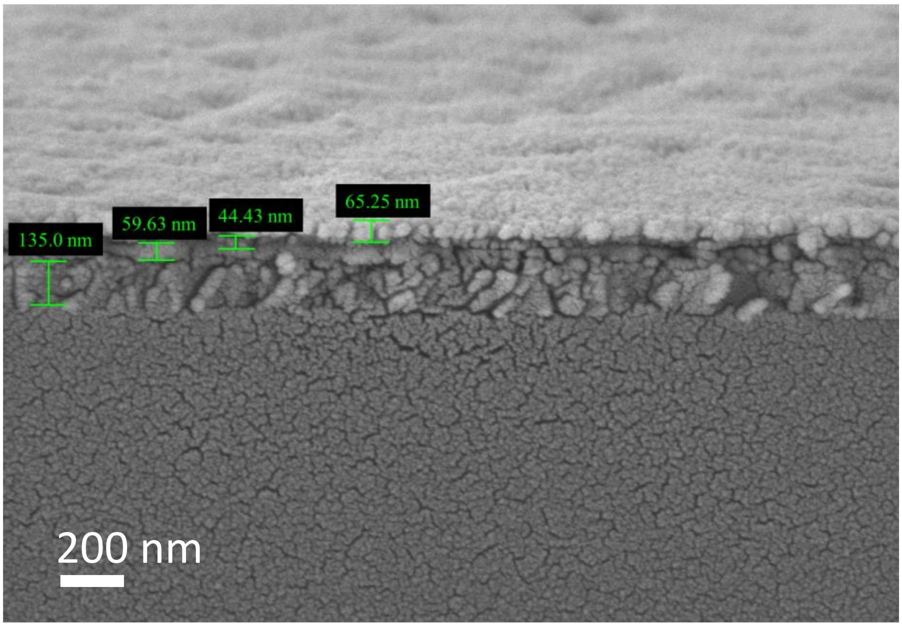

Figure S12. Cross-sectional SEM image of ZnMgO-based NPL-LEDs. The scale bar is 200 nm.

ZnMgO and NPL layers are difficult seperate from each other because both have a similar contrast under SEM. However, according to the concentration and spin-coating speed of ZnMgO, the thickness of ZnMgO is estimated to be ~30 nm. Therefore, the thickness of NPLs is also around ~30 nm. CBP and Al layers are challenging to separate because the thickness of MoO_3 is too thin. The estimated thickness is 6 nm for MoO_3 and ~60 nm for Al.

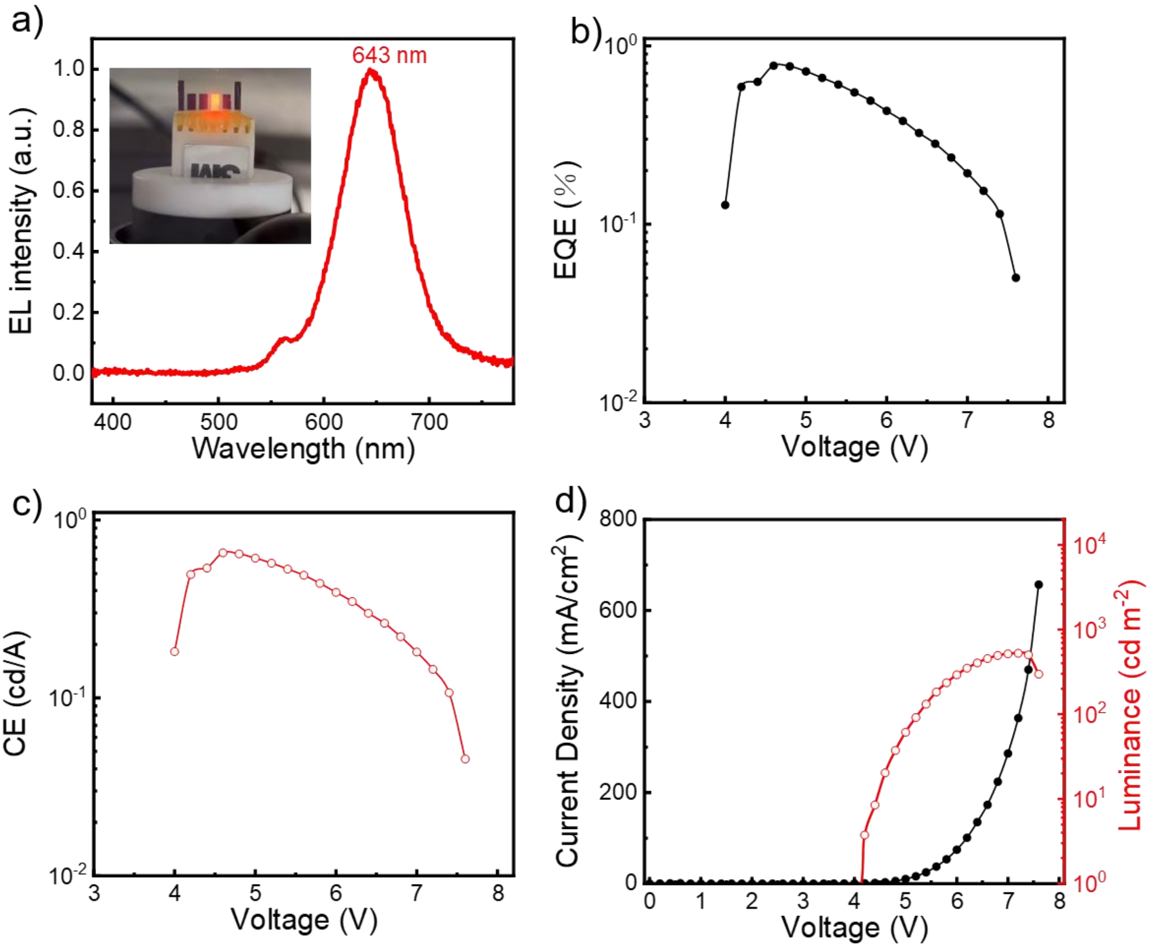

Figure S13. a) EL spectra of the core/crown NPL-based LEDs. Inset is a photograph of NPL-LEDs under bias. b) EQE, c) CE and d) current density and luminance of the core/crown NPL-based LEDs.

The maximum EQE and CE of the core/crown NPLs-LEDs are 0.77% and 0.65 cd A^{-1}, respectively, which are smaller than those of the multi-crowned NPL-based LEDs. In addition, the maximum luminance of the core/crown NPLs-based LEDs is 526 cd m^{-2}, and the turn-on voltage is 4.2 V.

Table S2. Summary of the performance of various type-II NPL-LEDs.

Structures	V_{on} (V)	EQE_{max} (%)	L_{max} (cd m^{-2})	λ (nm)	Ref.
$CdSe/CdSe_{0.8}Te_{0.2}$ core/crown	1.9	3.57	34,520	599	1
CdSe/CdTe/CdSe core/crown/crown	1.65	0.01	100	~650	2
CdSe/CdTe/CdSe core/crown/crown	3.0	7.83	10,765	647	This work

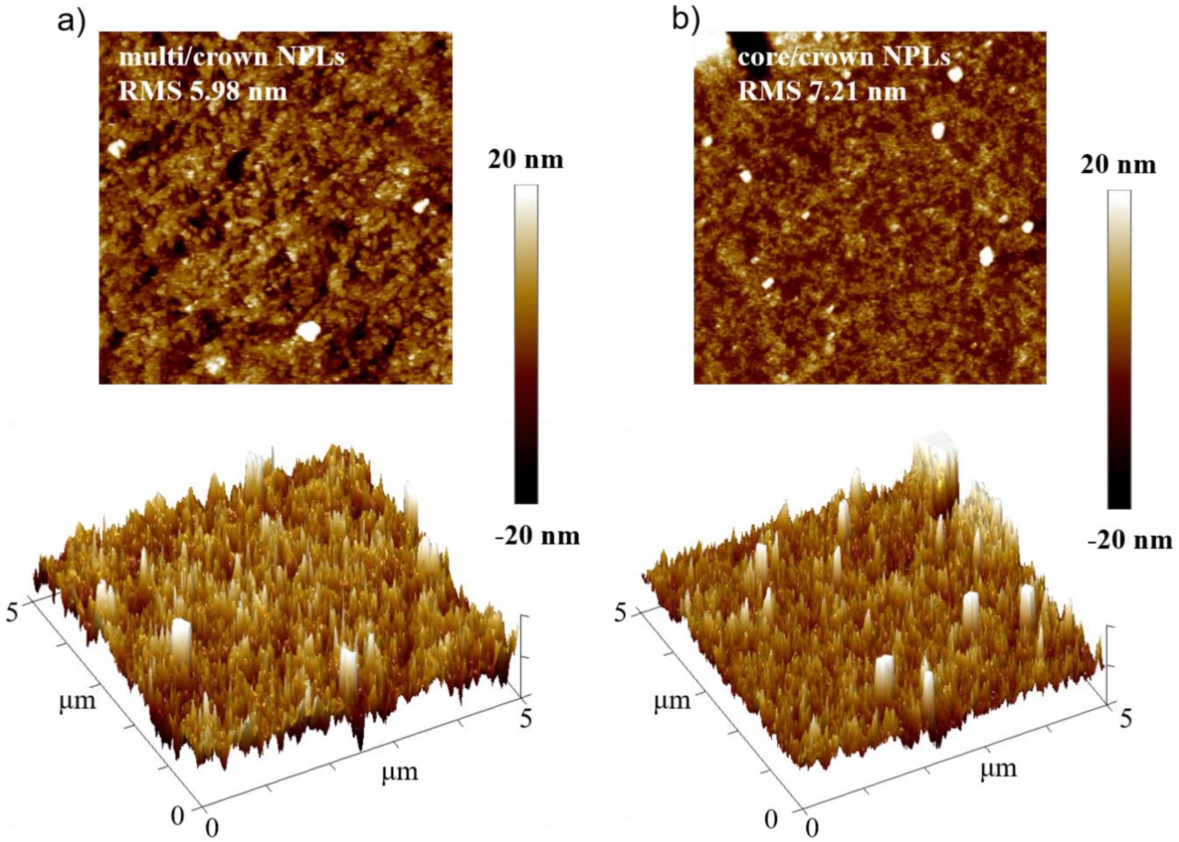

Figure S14. a) Atomic force microscopy (AFM) images for the multi-crowned NPL films (RMS=5.98 nm) and b) the core/crown NPL film (RMS=7.21 nm).

Theoretical Section

Wavefunction distribution calculations: We calculate the electron and hole excited state energies in the core/crown and multi-crowned NPLs considering the problem of a particle in a 2D box and solve the stationary Schrödinger equation (S2), assuming the effective mass approximation, using COMSOL Multiphysics:

$$\left[-\frac{\hbar^2}{2m_{e(h)}}\nabla^2_{e(h)}+V_{e(h)}\left(x,y\right)\right]\Psi_{e(h)}\left(x,y\right)=E\Psi_{e(h)}\left(x,y\right) \quad \text{(S2)}$$

Here $V_{e(h)}\left(x,y\right)$ is the potential arising from the conduction and valance band in the *x-y* plane for the electron (hole); $m_{e(h)}$ is the effective mass of the electron (hole), and $\Psi_{e(h)}$ denotes the envelope electron (hole) wavefunction.

The electron and hole excited energies can be calculated by considering the boundary and normalization conditions. In the case of multi-crowned NPLs, we assume the potential according to Figure S12a, where results from the conduction and valence band offset, 0.53 eV[3] and 0.63eV[3], between CdSe and CdTe, respectively, and the potential offset between the CdSe/ligand was taken as 2 eV.

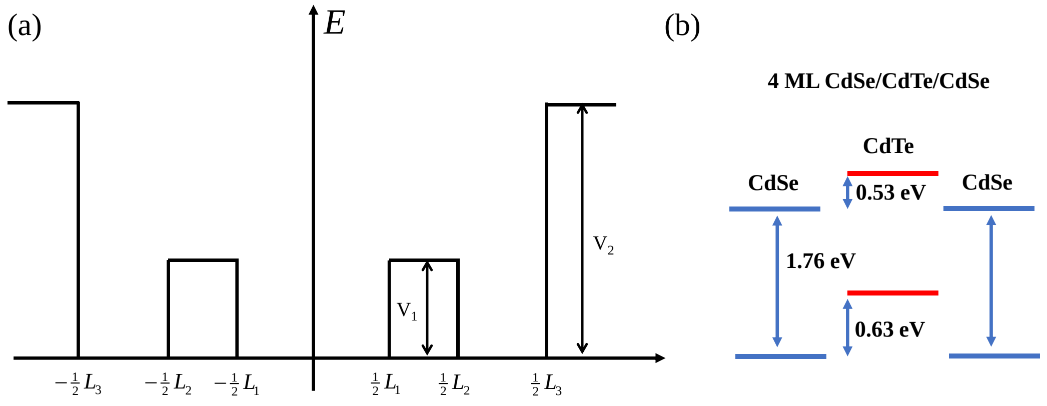

Figure S15. a) Potential diagram for the electron and hole in the multi-crowned NPLs. V_1 is the potential barrier offset between CdSe/CdTe for the electron and hole. V_2 represents the

potential barrier offset (*2 eV*) between the CdSe/ligand. (b) Potential barrier offsets for CdSe/CdTe/CdSe core/crown/crown heterostructure.

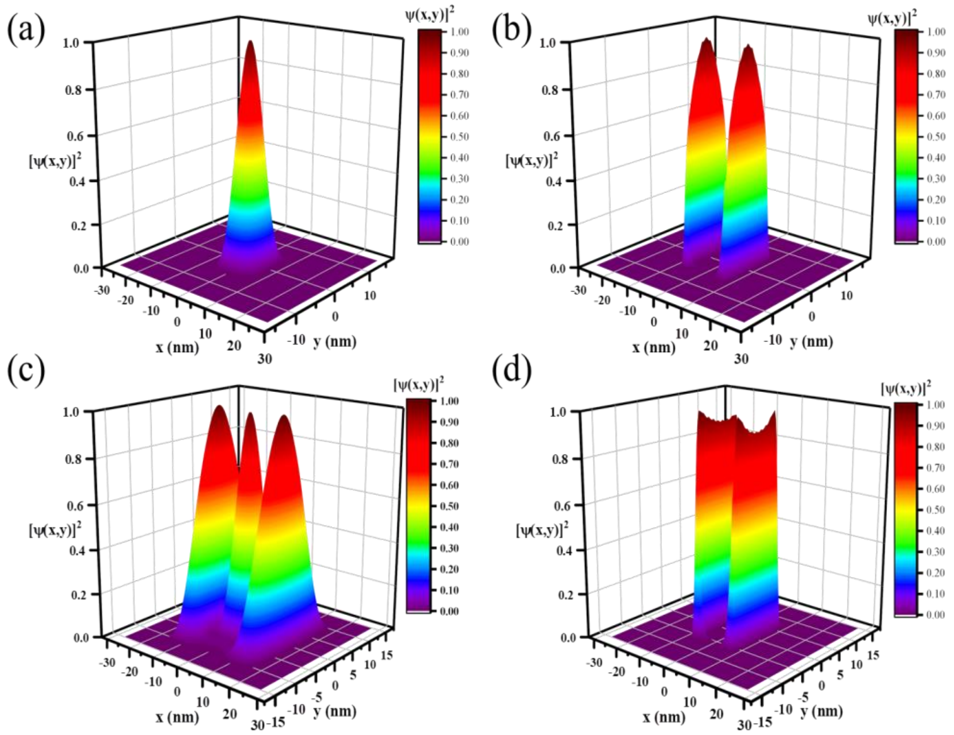

Figure S16. 3D electron wavefunction for a) the core/crown and c) the multi-crowned NPLs. 3D hole wavefunction for b) core/crown and d) multi-crowed NPLs.

Numerical Results: Table S3 lists the parameters used to calculate the electron and hole wavefunction of the core/crown and multi-crowed NPLs.

Table S3. List of parameters for CdSe and CdTe. m_0 is the electron rest mass. A thickness of 0.35 nm is assumed per ML.

Parameters	CdSe[3]	CdTe[3]
Electron effective mass	0.13 m_0	0.09 m_0
Hole effective mass	0.90 m_0	0.49 m_0

Reference

(1) Liu, B.; Delikanli, S.; Gao, Y.; Dede, D.; Gungor, K.; Demir, H. V. Nanocrystal Light-emitting Diodes based on Type II Nanoplatelets. *Nano Energy* **2018**, *47*, 115-122.
(2) Dabard, C.; Guilloux, V.; Greboval, C.; Po, H.; Makke, L.; Fu, N.; Xu, X. Z.; Silly, M. G.; Patriarche, G.; Lhuillier, E.; et al. Double-crowned 2D Semiconductor Nanoplatelets with Bicolor Power-tunable Emission. *Nat. Commun.* **2022**, *13* (1), 5094.
(3) Khan, A. H.; Bertrand, G. H. V.; Teitelboim, A.; Sekhar, M. C.; Polovitsyn, A.; Brescia, R.; Planelles, J.; Climente, J. I.; Oron, D.; Moreels, I. CdSe/CdS/CdTe Core/Barrier/Crown Nanoplatelets: Synthesis, Optoelectronic Properties, and Multiphoton Fluorescence Upconversion. *ACS Nano* **2020**, *14* (4), 4206-4215.
